# The co-occurrence of SAT, hypophysitis, and Schnitzler syndrome after COVID-19 vaccination: the first described case

**DOI:** 10.1007/s42000-024-00567-6

**Published:** 2024-05-22

**Authors:** Michał Szklarz, Katarzyna Gontarz-Nowak, Aleksander Kieroński, Krystian Golon, Jan Górny, Wojciech Matuszewski, Elżbieta Bandurska-Stankiewicz

**Affiliations:** 1 Clinic of Endocrinology, Diabetology and Internal Medicine, School of Medicine, Collegium Medicum, University of Warmia and Mazury in Olsztyn, 10-957 Olsztyn, Poland; 2 Department of Nuclear Medicine, School of Medicine, Collegium Medicum, University of Warmia and Mazury in Olsztyn, 10-957 Olsztyn, Poland; 3 Department of Pathomorphology and Forensic Medicine, School of Medicine, Collegium Medicum, University of Warmia and Mazury in Olsztyn, 10-957 Olsztyn, Poland

**Keywords:** ASIA syndrome, Subacute thyroiditis, Hypophysitis, Schnitzler syndrome, COVID-19, Vaccine

## Abstract

Subacute thyroiditis (also known as granulomatous thyroiditis, giant cell thyroiditis, de Quervain's disease, or SAT) is an inflammatory disease of the thyroid gland, usually spontaneously remitting, that lasts for weeks to months. However, recurrent forms sometimes occur which may have a genetic basis. In our paper, we have focused on the pathogenetics, symptoms, and treatment of SAT. We have described the 17-month disease course of a woman with persistent recurrent steroid-resistant SAT. SAT was well established and the patient's symptoms were not only recurrent neck pain with fever, but also recurrent chronic urticaria, which are symptoms that fulfil the criteria for the diagnosis of Schnitzler syndrome. Schnitzler syndrome occurred after vaccination with COVID-19 in the mechanism of ASIA syndrome. In our patient, Schnitzler syndrome involved the thyroid gland, causing persistent subacute thyroiditis, and the pituitary gland, causing transient swelling of the pituitary, which, to our knowledge, is the first reported case in the literature. Also unprecedented, as far as we know, is the fact that we performed thyroidectomy in the above patient, which reduced systemic inflammation and caused SAT to resolve, although only the inclusion of anakinra treatment resulted in resolution of the underlying condition.

## Background

Subacute thyroiditis (also known as SAT, granulomatous thyroiditis, giant cell thyroiditis, and de Quervain’s thyroiditis) is an inflammatory disease of the thyroid, usually spontaneously remitting, that lasts for weeks to months [1]. It involves diffuse neutrophilic invasion of the thyroid gland with active destruction of follicles and presence of multinucleated giant cells, fibrosis, and near complete loss of follicles. SAT was first described in 1895 by Mygind who published a report of 18 patients with "thyroiditis acuta simplex" [1]. The pathogenesis of SAT was discovered in 1904 by the Swiss surgeon, Fritz de Quervain. Although today SAT may account for 5% of all thyroid dysfunction cases [1], its incidence is still on the increase [2]. The incidence of SAT is 4.9 per 100,000 population, with a female to male ratio of 4:1 to 7:1 [3]. It is most prevalent in middle-aged women, who comprise up to 80% of all SAT patients [4]. Indirect evidence suggests that SAT may be triggered by a respiratory or gastrointestinal infection that occurs 2–6 weeks before the onset of SAT in genetically susceptible patients [5]. Prodromal symptoms of SAT usually include muscle pain, weakness, and fever.

## Signs and symptoms of SAT

The diagnosis is based on an elevated ESR and tenderness of the thyroid gland on palpation. Most commonly, the disease manifests with anterior neck pain that radiates to the ear, jaw, and down to the upper chest: pain has been reported by 93.75% of patients [4]. The pain is aggravated by head movements, coughing, or swallowing. The pain location may change over time, reflecting the progression of the disease. The condition may affect one thyroid lobe or, alternately, both lobes when the inflammatory process continues. However, in long-lasting thyroiditis, the whole thyroid gland tissue becomes hardened [6]. Moreover, patients complain of fatigue and of fever up to 39 °C, rising mostly in the evening and at night, this being observed in 65.6% of patients [4]. Laboratory test results show elevated ESR (43 mm/h on average) and CRP (which is considered less specific). Furthermore, a high white blood cell count, normocytic anemia, and increased levels of ferritin, IL-6, soluble intercellular adhesion molecules 1 [7–10], alkaline phosphatase (ALP), and liver enzyme are observed [11]. Usually, an increase in thyroid hormone levels is also seen. Thyroid peroxidase antibodies (anti-TPO) were detected in 4–15.5% of patients, thyroglobulin antibodies (anti-Tg) in 20–33%, and TSH receptor antibodies (TRAb) in 6% [4, 12]. Microhaematuria occurred in 63% of patients [4]. Thyroid ultrasound scans show central hypoechoicity, no microcalcifications, heterogenicity with ill-defined regions of poorly vascularized parenchyma, and characteristic "lava-flow” areas [13, 14]. On elastography, increased stiffness of the thyroid gland is typical [15]. SAT diagnosis criteria proposed by Lewiński et al. include elevated ESR or CRP, typical thyroid image on ultrasound, SAT-confirming or cancer-ruling out biopsy result as well as one of the following: hard thyroid swelling, pain and tenderness of the thyroid gland, elevated T4 with suppressed TSH, and decreased radioactive iodine uptake (RAIU). The decrease in RAIU is caused by thyrocyte destruction, which results in impaired iodine uptake [6]. The increase in TRAb, which may sometimes cause diagnostic problems, is the consequence of the inflammation-induced B lymphocyte activation; however, the coexistence of TRAb and SAT does not affect the course of hyperthyroidism in SAT [4]. Anti-Tg are usually transiently elevated because of the enhanced antigen presentation in the active phase of the disease, while anti-TPO elevation may be persistent and increase the risk for autoimmune thyroid disease (AITD) in the future [4]. In most cases, the disease progression can be divided into three phases, starting from the phase of hyperthyroidism due to thyroid gland destruction, and followed by the phase of pain alleviation and, subsequently, the phase of transient hypothyroidism of 1–2 months’ duration [4]. Although in some patients the disease can resolve spontaneously within 8–16 weeks, most cases require pharmacological treatment. The hyperthyroid phase may last 3–4 months and usually manifests with mild symptoms because of relatively low fT3 levels (T4:T3 ratio is usually > 20) [16].

## Genetic susceptibility to SAT

The occurrence of SAT is associated with genetic predisposition encoded in the sixth chromosome fragment responsible for the HLA tissue compatibility antigen system. Susceptibility to the disease coexists with specific haplotypes, while, most often, it is the presence of certain alleles that determines the clinical course of the disease. Nyulassy et al. in 1975 described for the first time an increased prevalence of HLA-B35 in patients suffering from SAT [17]. Several other authors have found that the prevalence of HLA-B35 ranges from 67 to 72.5% in patients with SAT [18, 19]. In other studies, the presence of HLA-C*04:01 was described in 75.5% of patients with SAT versus 12.5% in the control group [20]. Ohsako et al. reported higher prevalence of HLA-Bw67 and HLA-Cw in their group of patients with SAT [19]. Robin and Guay et al. confirmed the correlation between SAT and HLA-Cw4 [21]. In the Netherlands, atypical SAT was described in a group of patients in whom HLA-B15 and B62 were shown in 5/11 of the patients, including only one patient with positive HLABw35 [22]. HLA-C*04:01 is in linkage disequilibrium with HLA-B*35:01/02/03 [20]. The occurrence of HLA-B*35 and HLA-C*04:01 is a marker of genetic susceptibility to SAT. Nevertheless, C*04:01 cannot be perceived as the only risk factor. No linkage with B*35 has been described for B*18:01 or DRB*1:01, thus, these alleles should be considered completely independent risk factors for SAT. HLA-B*18:01 was found in 23.3% of patients with SAT versus 7.2% in the control group, while HLA-DRB1*01 was found in 28.3% of SAT patients versus 12.9% of the control group [20].

Genetically encoded differences in innate immunity may influence the development of SAT. HLA controlled complement components 3 and 4 are usually decreased in SAT [18]. HLA-linked genes could be involved in regulation of IgA and alpha 2 macroglobulin. B35 antigen can be referred to as a "virus susceptibility gene complex" because it is associated with a number of other diseases known to have viral pathogenesis, including Hodgkin’s disease [18], Schonlein-Henoch purpura [23], Balkan endemic nephropathy, and mononucleosis [24].

Evidence suggests a correlation between thyroid ultrasound image and the carrier state of specific alleles. Carriers of HLA-B35 and HLA-C04:01 were found to have hypoechoic heterogenous areas with blurred margins and decreased vascularization. HLA-DRB1:01 carriers showed a thyroid sonographic pattern typical of SAT. Patients with HLA-B*18:01 presented unilateral hypoechoic homogenous lesions that involved the whole thyroid lobe and mimicked large thyroid nodules. Vascularization was reduced or absent in these patients. The lesions were unilateral in B18:01 carriers, but carriers of B18:01 and B35 were more likely to have bilateral lesions [14].

## Recurrent SAT

The recurrence rate of SAT ranges from 1.6% to 20% [25, 26]. The recurrence may occur immediately after the therapy discontinuation or up to several years after the first episode [27]. SAT recurrences 6–21 years after the first episode were reported in up to 4% of patients [3]. Some patients may become steroid dependent and any attempt to discontinue the therapy in them results in SAT relapse. The reasons for SAT recurrences are not clear but the risk of SAT recurrence is known to be HLA-dependent. Although Yamamoto et al. proposed a correlation between HLA-A*26 and SAT recurrences, their findings have not been confirmed [27]. SAT recurrence depends on co-presence of HLA-B*18:01 and B35 [2]. SAT was found to recur in 44.4% of patients with co-presence of 18:01 and B35 versus 5% of patients without these two haplotypes. Co-presence of HLA-DRB1*15:01 and/or B*07:02 along with absence of HLA-A*01:01 and B*41:01 seemed to be key factors protecting from recurrent SAT and steroid dependence [5].

Factors that protected against SAT recurrence were biochemical thyrotoxicosis and positive anti-TPO titers [2]. This suggests that more pronounced thyroid destruction and concomitant AITD can protect against the repeat SAT attack. A meta-analysis of 18 cohorts has shown that increased thyroid volume and further extension of the hypoechoic area are positively correlated with SAT recurrences [28]. In a study of 137 patients by Bahadir et al., a group with recurrent SAT presented higher TSH levels, lower fT4 levels, and lower ECR before the therapy initiation [29].

## SAT following COVID-19 infection and COVID-19 vaccination

SARS-CoV-2 is a multiorgan disorder due to ubiquitous expression of ACE-2, this being a functional receptor through which the virus enters the human cells [30, 31]. SARS-CoV-2 has significant tissue tropism, including a high affinity for thyroid tissue. ACE-2 mRNA was detected in thyroid follicular cells, which enables the virus to enter the thyrocytes [32]. SAT may be triggered either by SARS-CoV-2 infection or by SARS-CoV-2 vaccination. A total of 10–20% of patients hospitalized for COVID-19 reported symptoms typical of SAT [33]. Although the available body of evidence suggests a very favorable safety profile of COVID-19 vaccines [30], we must remember that reporting side effects, including side effects of vaccines, is obligatory for every doctor. Adjuvants are added to vaccines to enhance the immune response to the vaccination. A possible mechanism proposed as possibly being involved in SAT occurrence after the SARS-CoV-2 vaccination is ASIA syndrome. Genetically predisposed patients may develop ASIA syndrome as a result of an immune disorder, molecular mimicry, and polyclonal B cell activation [34]. The ASIA syndrome, described for the first time in 2011, was reported following the vaccinations against HPV, HBV, and influenza [35]. Vaccination-induced ASIA syndrome may lead to endocrinopathies including SAT [34]. Moreover SARS-CoV-2 spike protein, nucleoprotein and cell membrane protein are all elements that can cross-react with TPO, which may share some protein sequences with SARS-CoV-2 proteins [36]. Generation of SARS-CoV-2 antibodies may promote thyroid autoimmunity. The female patient reported in our study has a haplotype associated with increased genetic susceptibility to ASIA syndrome following SARS-CoV-2 vaccination. In a study of 14 patients with post-vaccination SAT compared to 100 control patients, the presence of HLA-B35 and HLA-C*04:01 alleles was considerably more prevalent in patients with SAT induced by SARS-CoV2 vaccination [37].

## Urticaria and AITD

Urticaria is a sudden appearance of erythematous skin lesions with or without accompanying angioedema of the deeper skin layers [38]. Chronic urticaria/angioedema (CUA) is defined as the presence of skin lesions for more than 6 weeks. CUA most commonly affects people between their 3rd and 5th decades of life with a female to male ratio of 2:1 and the prevalence of 0.3–1% in the general population [39, 40]. CUA is combined with autoimmunity in 30–45% of cases [41]. A correlation between CUA and autoimmune disorders was demonstrated by Chiu who showed that they may share genetic pathways [42]. Leznof was the first to report that 12.1% of CUA patients suffer from concomitant Hashimoto disease [43]. Positive anti-TPO and anti-Tg antibodies were associated with higher prevalence of antibodies against the IgE receptor [44]. Higher prevalence of anti-TPO IgE was shown in CUA patients versus the control group [45]. Anti-TPO IgE antibodies are able to activate basophils and may induce wheals in the skin in the course of chronic spontaneous urticaria (CSU) [45]. Turkoglu et al. showed that the autologous serum skin test (ASST) was more frequently positive in patients with AITD, which suggests increased autoreactivity of mast cells in these patients [46]. Silvares et al. observed that female patients with CUA and concomitant AITD presented more pronounced response in ASST and higher TSHR expression in the skin [47]. In 2011 Gulec et al. discovered a potential relationship between thyroid inflammatory infiltration and the occurrence of urticaria. Additionally, an intervention involving L-thyroxine administration resulted in the reduction of neopterin urine levels and alleviation of urticaria [48]. Urticaria, like any other uncommon severe allergic adverse reaction, can develop after COVID-19 vaccination. There are two main allergens in the COVID-19 vaccine, namely, PEG and polysorbate 80 [49]. Coincidence of CUA and SAT in a single patient appears to be unusual. Both diseases can be induced by viral infections mediated by cytokines with impaired T cell response. Urticaria can therefore develop as a hypersensitivity reaction to systemic inflammation triggered by SAT. The following case report was found in the literature: A 45-year old patient with recurrent idiopathic urticaria and swollen mouth had no more CUA recurrences at 12month follow-up after having his SAT cured [50]. HLA alleles may be involved in the pathogenesis of CUA and they appear to be directly involved in the initiation of immune response [51]. The female patient described in our case report is genetically at higher risk of CUA, being a carrier of HLA-DRB1*01:01 and HLA-DQ1 alleles. Dogan et al. confirmed that the presence of HLA-DRB1 increased the risk of CUA [52], while other authors indicated that HLA-DRB1:01, HLA-Bw4, HLA-DQ1, and HLA-DRB*15 were predominant alleles in the patient group with CUA [53, 54].

### Schnitzler syndrome

Schnitzler syndrome is a rare acquired autoinflammatory syndrome described for the first time in 1972 by Liliane Schnitzler [55] and characterized by chronic urticarial rash and immunoglobulin M monoclonal gammopathy of undetermined significance (MGUS), these being the two essential features required for diagnosis. As the disease entity is very infrequent, its diagnosis is often delayed by several years. However its recognition is of key importance because of the risk for serious secondary conditions induced by chronic inflammation. Until now, only a few hundred cases of Schnitzler syndrome have been reported worldwide, with no cases of concomitant Schnitzler syndrome and SAT, and one case of an 86-year old woman with Schnitzler syndrome following COVID-19 vaccination [56]. The disease can lead to the development of lymphoproliferative disease in 15–20% of patients and AA amyloidosis. The average age of disease onset is 55 years and it is found mostly in Caucasians, with an incidence rate of 53–71 new cases/million population/ year [57]. The pathogenesis of the syndrome is unclear. There is a body of evidence reporting that it is an acquired autoimmune disease. Schnitzler syndrome shares many similarities with cryopyrin associated periodic syndrome (CAPS), a genetically determined autoimmune disorder resulting from a mutation in the NLPR3 gene. No germinal mutation in the NLPR3 gene has been found, but somatic NLPR3 mutations have been reported in two patients with the IgG variant of Schnitzler syndrome [55]. There have been reports of increased IL-1 beta and IL-6 levels in Schnitzler syndrome [58], which might be produced by mast cells of the skin [58]. Overproduction of IL-1 can result in decreased lL-17 levels [59], while anti-IL-1 therapy can restore IL10. The role of IgM paraprotein remains unclear: it is not known whether IgM is the consequence or the cause of the disease. It is merely an increase in IL-1 secretion by My-D88 that results in IgM overproduction. Recurrent urticaria is observed in all patients, predominantly on the limbs and trunk. The eruptions consist of rose macules or raised papules or plaques and can be itchy. The lesions can be exacerbated by stress or exercise. They usually persist for 24 h with variable intensity. Sometimes dermographism and a halo of vasoconstriction can be seen. Skin biopsy should be taken from a recent lesion. The most typical biopsy results include neutrophilic urticarial dermatosis, perivascular and interstitial infiltrate of neutrophils with leukocytoclasia [59].

Recurrent fever, usually accompanying skin rash and arthralgia, is observed in most patients. Joint and bone pains occur in 40% of patients, mostly in the lower extremities. Hepato- and splenomegaly were also reported, as well as enlarged lymph nodes, body weight loss, myalgia, and headaches. Monoclonal IgM (MGUS) is observed in 88% cases [55], mostly associated with kappa light chain. Skeletal abnormalities are also observed, including an increased uptake on scintigraphy and sclerosis on X-ray.

Skin biopsy shows lymphocytic infiltrations. Strasbourg diagnostic criteria include obligate criteria, i.e., chronic urticarial rash and monoclonal IgM or IgG, and minor criteria, i.e., recurrent fever, objective finding of abnormal bone remodeling with or without bone pain, a neutrophilic dermal infiltrate on skin biopsy, leukocytosis, and/or elevated CRP. Definitive diagnosis can be made if two obligate criteria and at least two minor criteria if IgM and three minor criteria if IgG are met. Probable diagnosis is made if two obligate criteria and at least one minor criteria if IgM and two minor criteria if IgG are met [55, 59].

### Treatment of Schnitzler syndrome

In Schnitzler syndrome, anakinra represents an effective, verified, and safe medication with potentially long-term administration not compromising its original efficacy and subjective tolerance. Anakinra, which blocks autonomous inflammatory reaction of the organism via the interleukin 1 pathway due to an increase in IL-10 levels, is a generally accepted first line treatment. Therapy with anakinra results in complete and partial remissions in 83% and 17% of cases, respectively. Complete remission was characterized by urticaria and pain regression (within hours), normalization of inflammatory markers (within days), and bone metabolism improvement assessed by the markers of osteoblastic osteoformation and osteoclastic osteoresorption. With normalized inflammatory markers (including interleukin 6 and interleukin 18), arthralgia, and sporadic exacerbations of urticaria and fevers persist in the patient in partial remission [55].

## Treatment of recurrent SAT

Antithyroid medications are contraindicated. The first-line treatment consists of non-steroidal anti-inflammatory drugs (NSAID) or glucocorticoids (starting from prednisone 40 mg with subsequent dose tapering). Several studies suggest that in some cases, prednisone 15 mg daily as the initial dose may be sufficient [60]. Long-term glucocorticoid treatment is associated not only with Cushingoid appearance but in particular with metabolic complications, including diabetes, hyperlipidemia, and arterial hypertension, as well as with the development of osteoporosis, infections, acne, easy bruising, and depression. There are reports in the literature of successful treatment of recurrent SAT with radioactive iodine, but due to their scarcity and inconsistent results no final conclusions can be drawn [61].

Pharmacological treatment failed to be effective before thyroidectomy, although, importantly, this varied in different patients. Most patients were administered prednisone at daily doses of 10–30 mg, some patients received prednisone 40 mg daily (i.e., the recommended dose in SAT) [62], and one patient received prednisone 70 mg daily, however, with no success and with disease relapses at steroid discontinuation attempts [63]. It is important to remember that in patients at high risk of recurrence, the therapy should be started with a high dose of prednisone with further gradual dose reduction over a longer period of time. However, in our patient even this approach proved to be ineffective.

## Case report

The case discussed below involves a 52-year old female patient with recurrent SAT, hypophysitis, and concomitant Schnitzler syndrome in whom the triggering factor was SARS-CoV-2 vaccination.

**Fig. 1:**
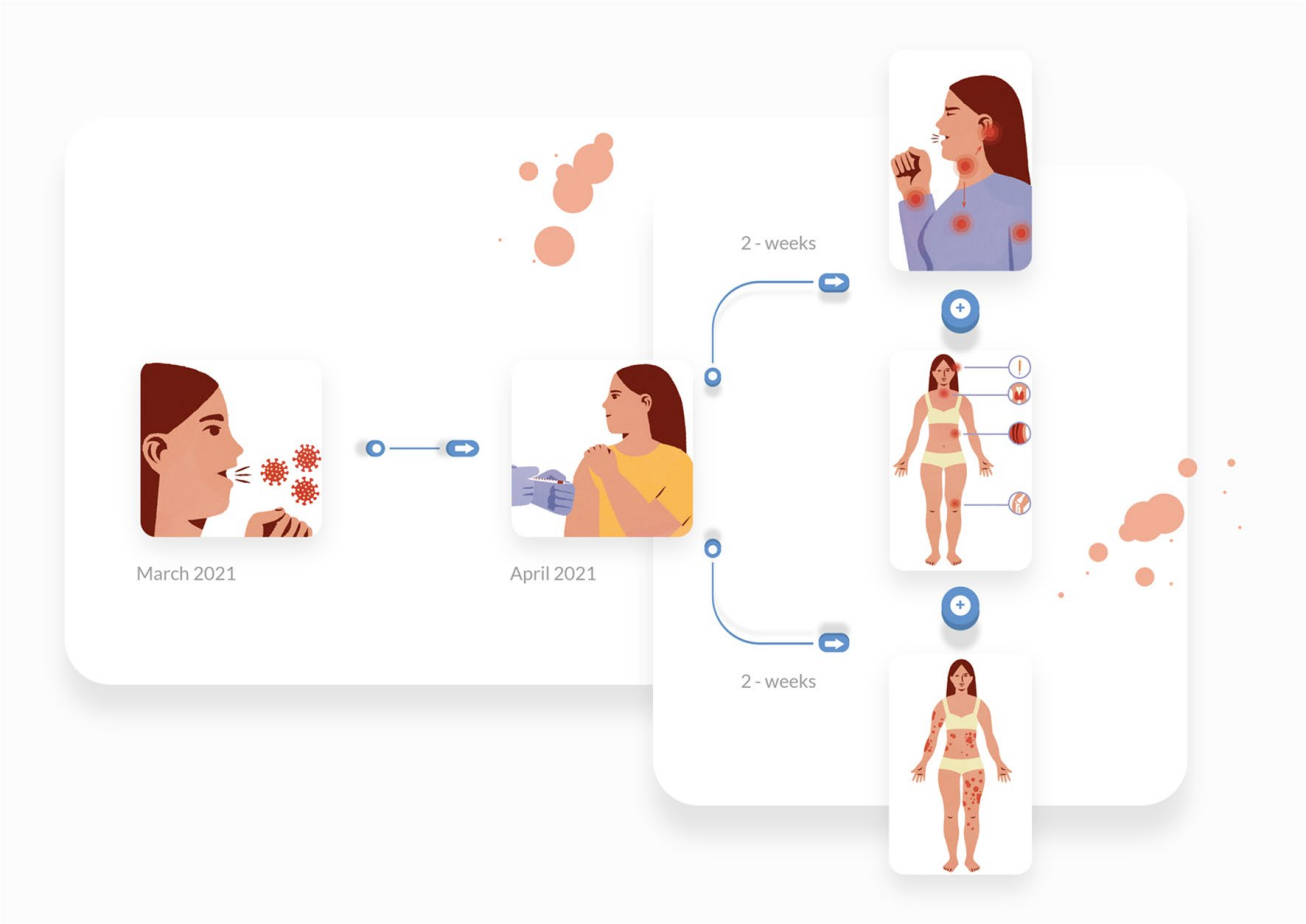
Disease pattern: post COVID-19 status in March 2021. Patient vaccinated against COVID-19 (BioNTech; Pfizer—Comirnaty) in April 2021. 2 weeks after the vaccination, skin lesions in the form of urticaria appeared, and after another week the first episode of subacute thyroiditis occurred

**Fig. 2:**
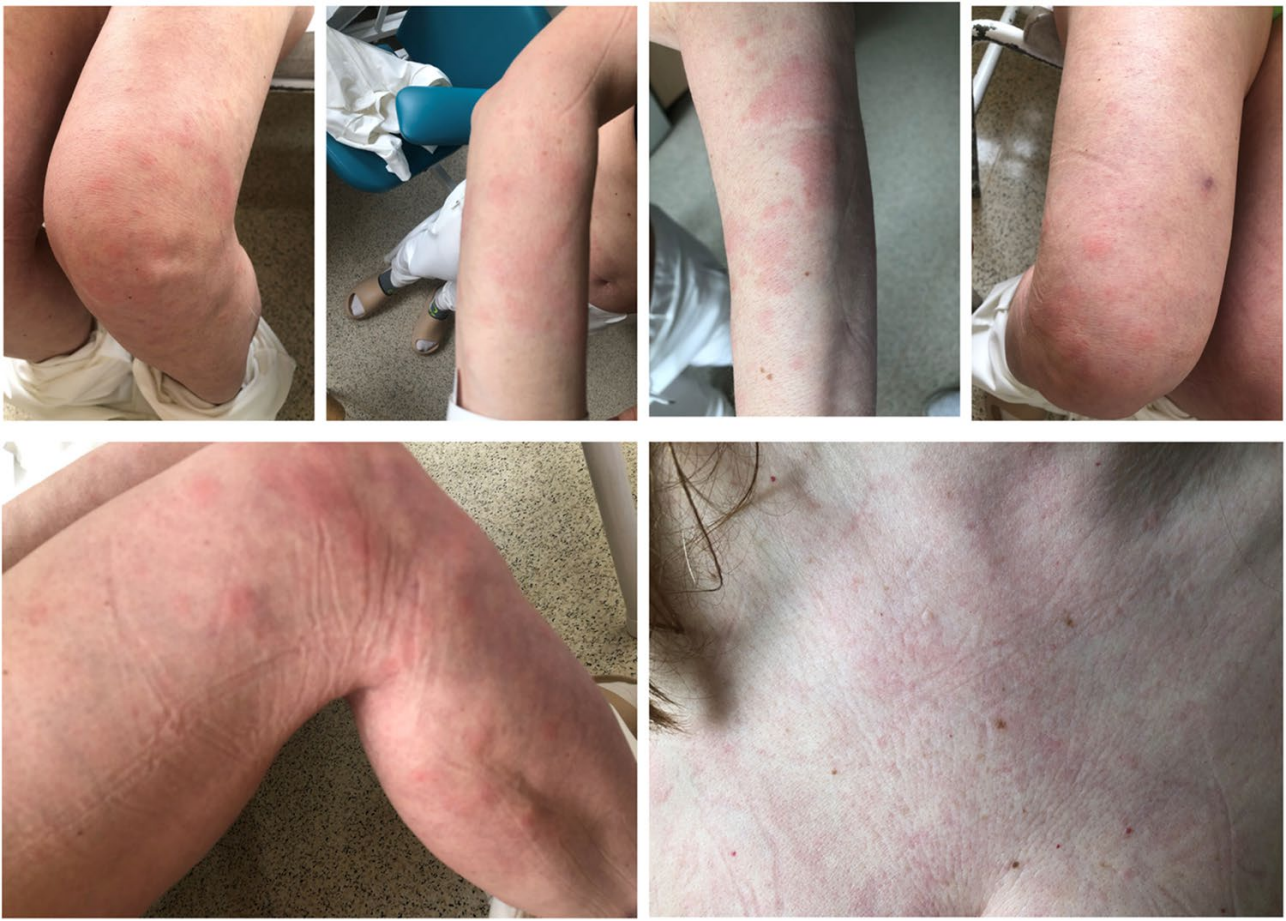
Urticarial lesions on the skin of the trunk and extremities

**Fig. 3:**
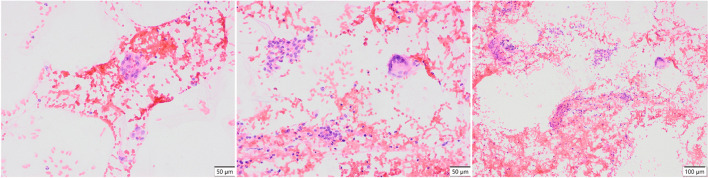
Fine-needle aspiration biopsy of the thyroid gland, HE Subacute thyroiditis lesion with prominent multinucleated giant cell type around a foreign body

**Fig. 4:**
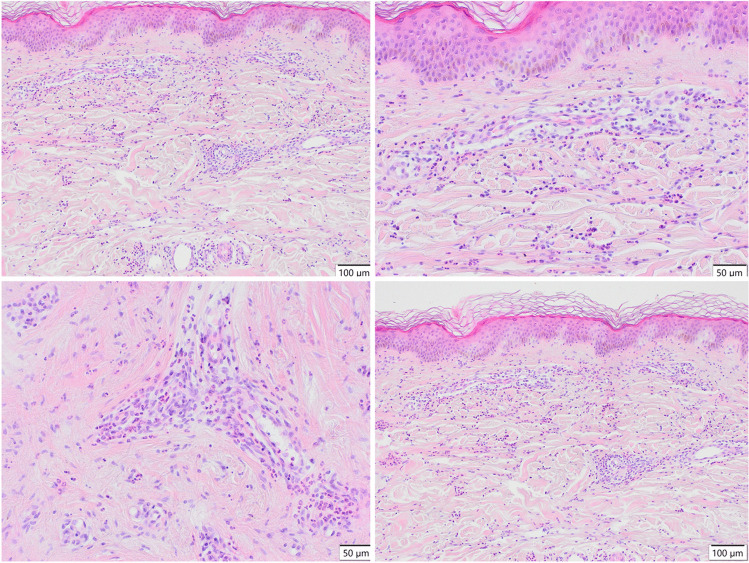
Skin biopsy from the right lower leg. HE. Changes consistent with urticaria: dilated blood vessels, features of edema in the dermis, perivascularly and between collagen fibers scattered acidophilic granulocytes and lymphoid cells

**Fig. 5:**
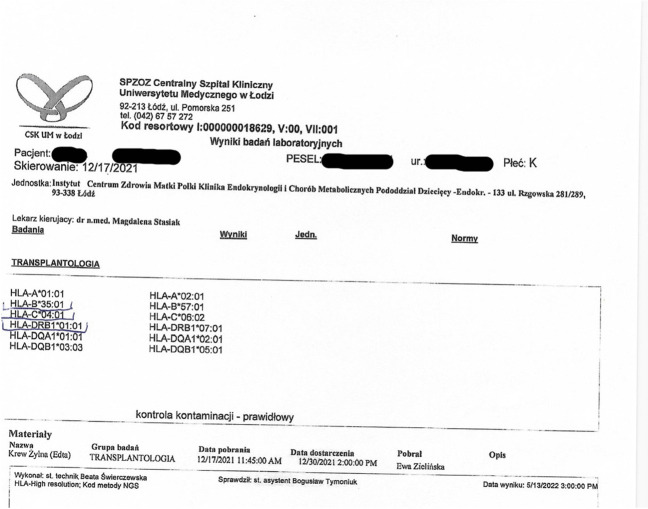
Genetic test result

**Fig. 6:**
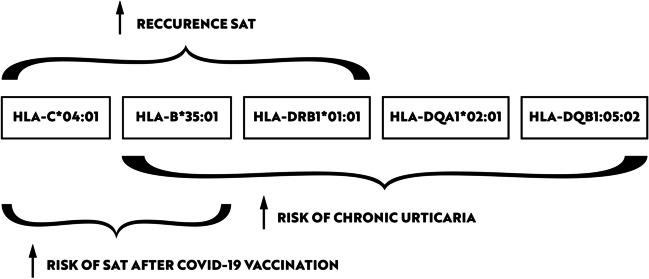
Diagram showing how the carrying of particular haplotypes in our patient could have affected the disease picture

**Fig. 7:**
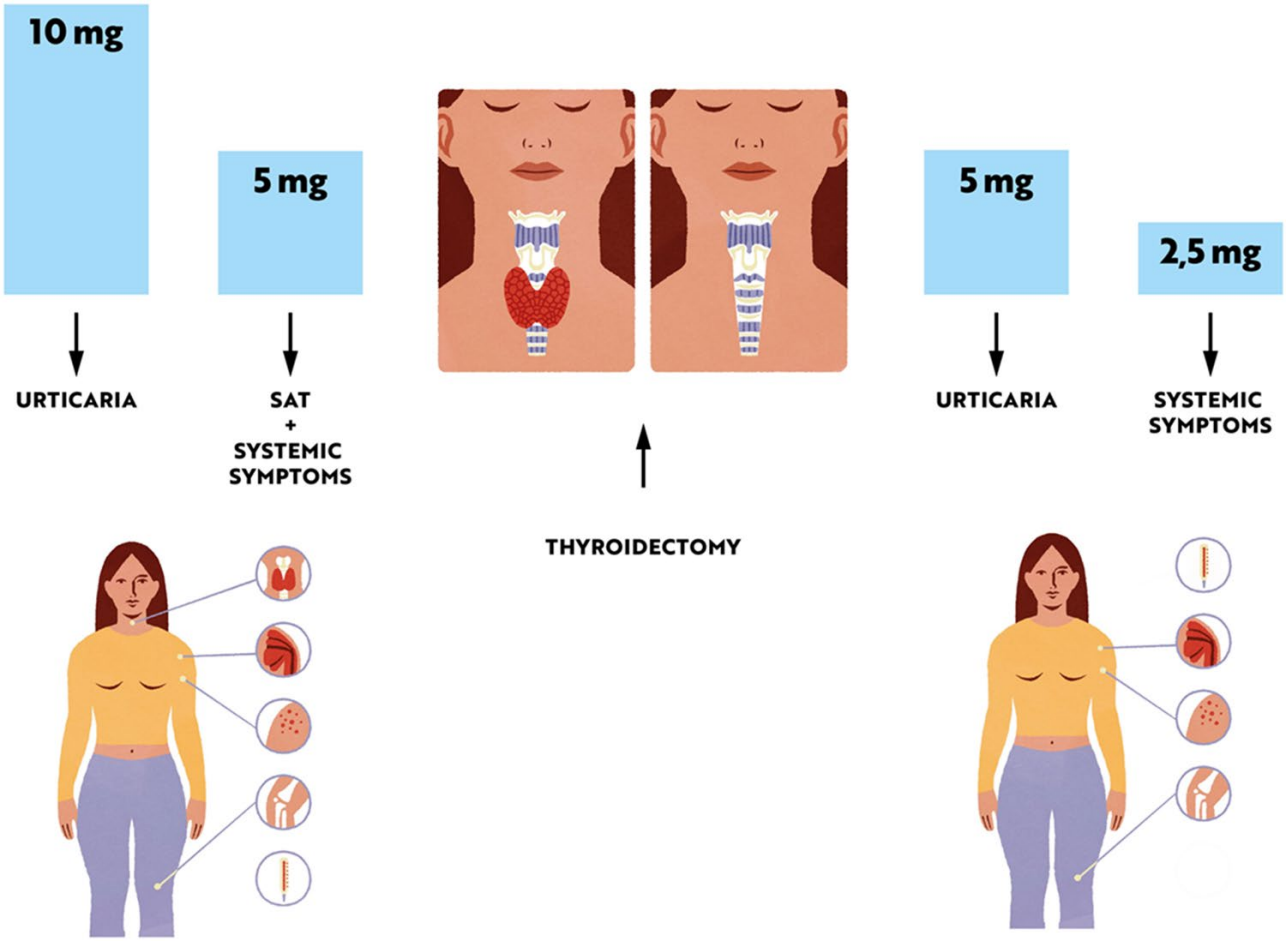
Diagram showing the effect of thyroidectomy. In the postoperative period, urticaria occurred after the prednisone dose was reduced to 5 mg (versus 10 mg in the preoperative period), and general symptoms occurred with further dose reduction to 2.5 mg (versus 5 mg in the preoperative period)

**Fig. 8:**
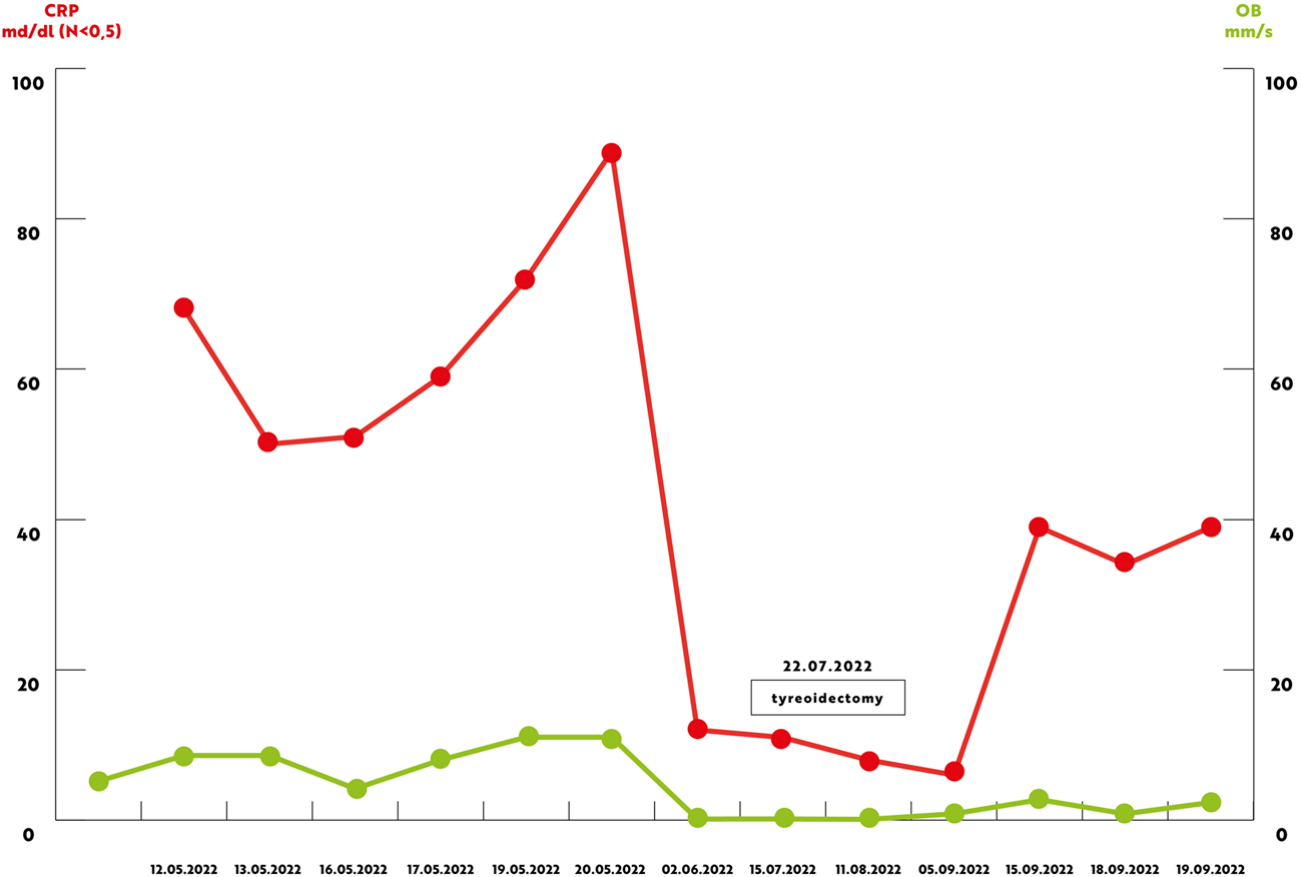
Graph showing the concentrations of the inflammatory markers (CRP and ESR) in the preoperative and postoperative periods

**Fig. 9:**
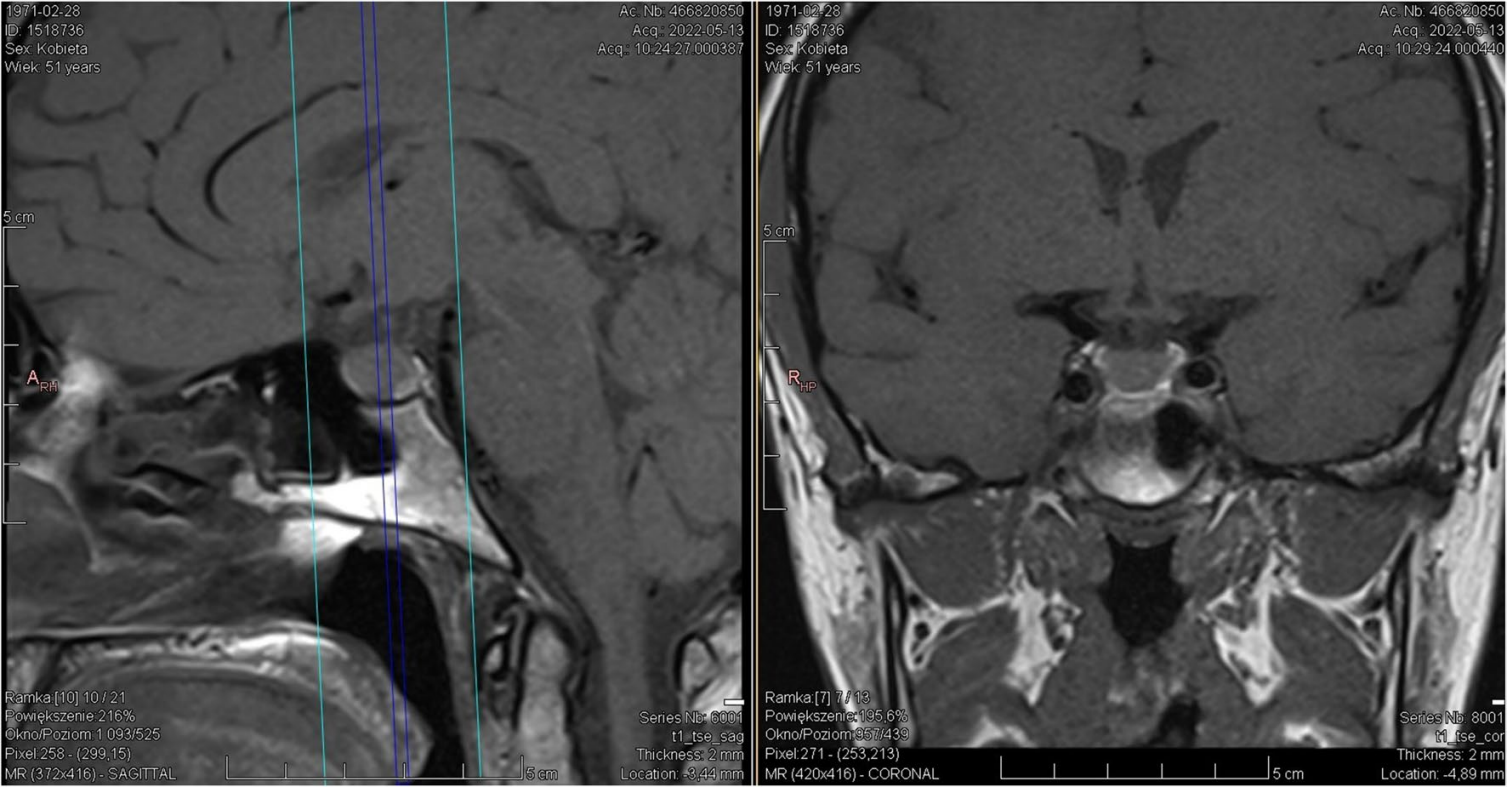
Transient pituitary oedema most likely in the course of the underlying disease

**Fig. 10:**
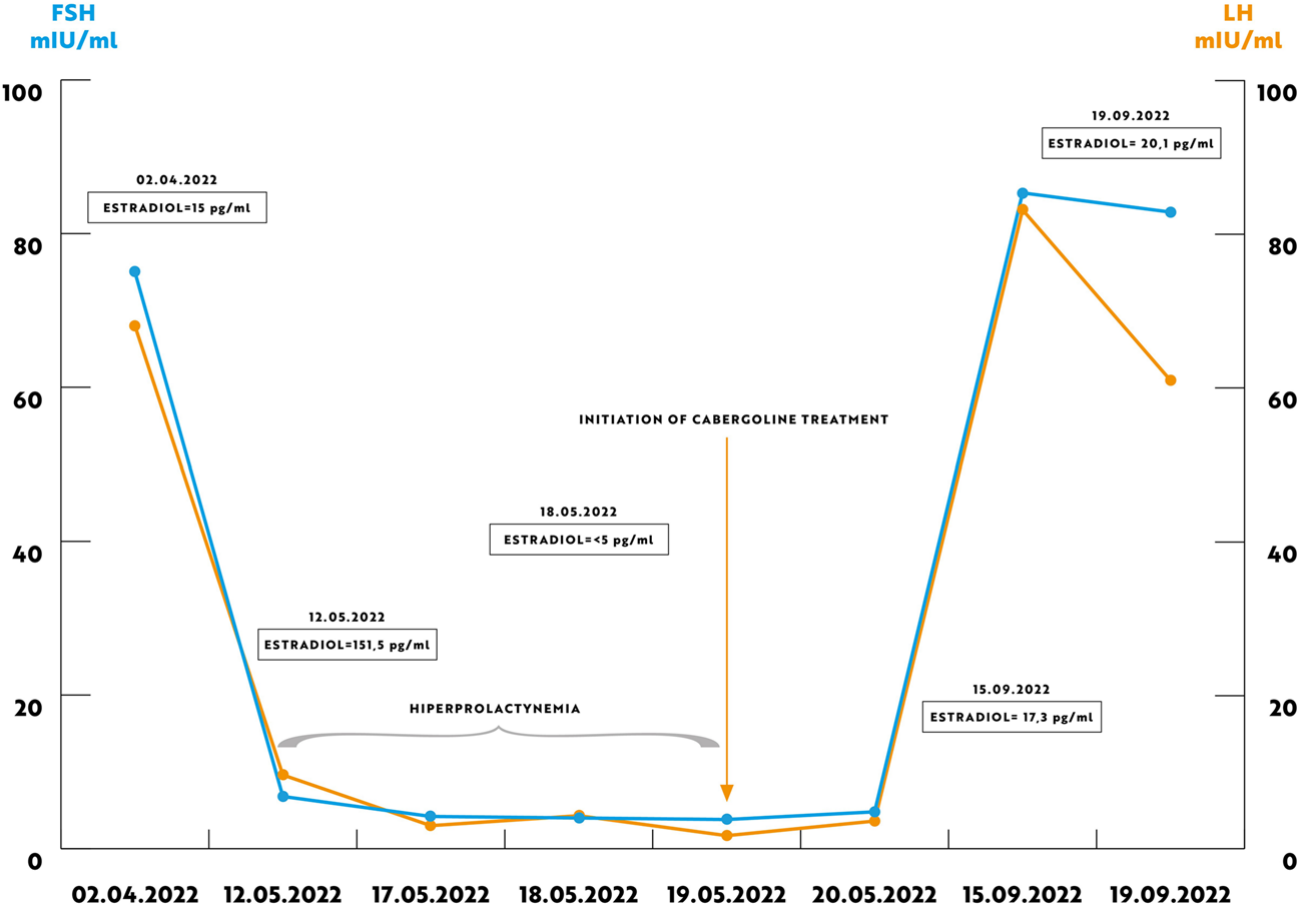
Graph showing the concentrations of gonadotropins, oestradiol and prolactin

We report a case of a 52-year old woman, 1 year after menopause, 3 months after SARS-CoV-2 infection, and 12 days after COVID-19 vaccination (BioNTech; Pfizer – Comirnaty). She presented with fever up to 38 °C, neck pain at palpation, and muscle and joint pains. The symptoms were accompanied by urticarial skin lesions on the trunk and limbs (Fig. 1 and 2) Based on her laboratory test results (CRP, ESR, and blood cell count), thyroid ultrasound scans, and fine needle aspiration (FNA) biopsy findings, the patient was diagnosed with subacute thyroiditis (SAT) and received therapy with prednisolone 40 mg daily, with immediate clinical improvement. Unfortunately, every attempt to withdraw the glucocorticoid therapy 6 weeks later resulted in recurrent SAT and urticaria, confirmed by FNA biopsy and histopathologic examination, respectively (Fig. 3 and 4.) Urticaria recurred when the prednisolone dose was reduced (down to 10 mg daily) and fever with neck pain was observed with further steroid dose reduction (down do 5 mg of prednisone daily). In total, the patient suffered from five SAT recurrences. It is of interest to note that thyroid hormone levels remained unchanged in all SAT episodes and thyroid antibodies were negative. During further follow-up, consecutive attempts to withdraw glucocorticoid therapy manifested with five relapses, confirmed by laboratory test results, ultrasound scans, and FNA biopsy findings. Genetic analysis revealed the presence of three alleles associated with a high risk of SAT development, specifically, HLA-B*35:01, HLA-C*04:01, and HLA-DRB1*01:01, which suggests high genetic susceptibility to SAT. However, the allele profile was not typical of a high risk of recurrent SAT. The presence of HLA-B*35:01, HLA-DQA1*02:01, and HLA-DQB1:05:02 alleles in our patient identify her as having increased susceptibility to chronic urticaria. The presence of HLA-B*35:01 and HLA-C*04:01 identifies increased genetic susceptibility to ASIA syndrome following SARS-CoV-2 vaccination (Fig. 5 and 6) Due to the multiple recurring episodes of SAT and based on the results of genetic and cytological examinations, the patient was found to be suitable for total thyroidectomy, which, in fact, is not the recommended treatment for SAT. The surgery was performed with no complications and the patient’s thyrometabolic status was subsequently normal with continued L-thyroxine replacement therapy at a dose of 88 mcg. The thyroid resection resulted in relief of neck pain, but fever, muscle pain, and urticaria recurred when glucocorticoid therapy was withdrawn. The prednisone dose reduction down to 5 mg daily caused the recurrence of urticaria, and with further prednisone dose reduction down to 2 mg daily, recurrence of fever was observed (Fig. 7 and 8). Interestingly, it was already during the treatment of recurrent episodes of SAT that FSH and LH decreased and hyperprolactinemia occurred, with pituitary MRI scans showing the features of transient pituitary edema (Fig. 9), accompanied by headache. Within 4 months of cabergoline and corticosteroid treatment, the edema was resorbed, prolactin levels returned to normal, and FSH and LH levels returned to postmenopausal values (Fig. 10), while the complaints of headache have subsided. The main criteria that led us to the diagnosis of hypophysistis were increased prolactin levels and recurrent headache. Elevated prolactin levels have been described in 30% of hypophisitis patients and have been reported in lymphocytic hypophystitis in men and non-pregnant women, most likely as a result of compression of the pituitary stalk [64]. Headache is the most common symptom of hypophysitis, affecting 60% of hypophysitis patients. In hypophysitis, the gland is generally symmetrically enlarged, with administration of gadolinium homogeneously enhancing the gland. Furthermore, in hypophysitis, the pituitary displays a relatively low signal on T-1 and a relatively high signal on T-2 weighted images. In contrast, in adenomas, gadolinium enhances the gland more focally. Finally, we did not detect any lesions typical of sarcoidosis, histiocytosis, Wegener’s granulomatosis, or IgG4-related hypophysitis.

**Fig. 11:**
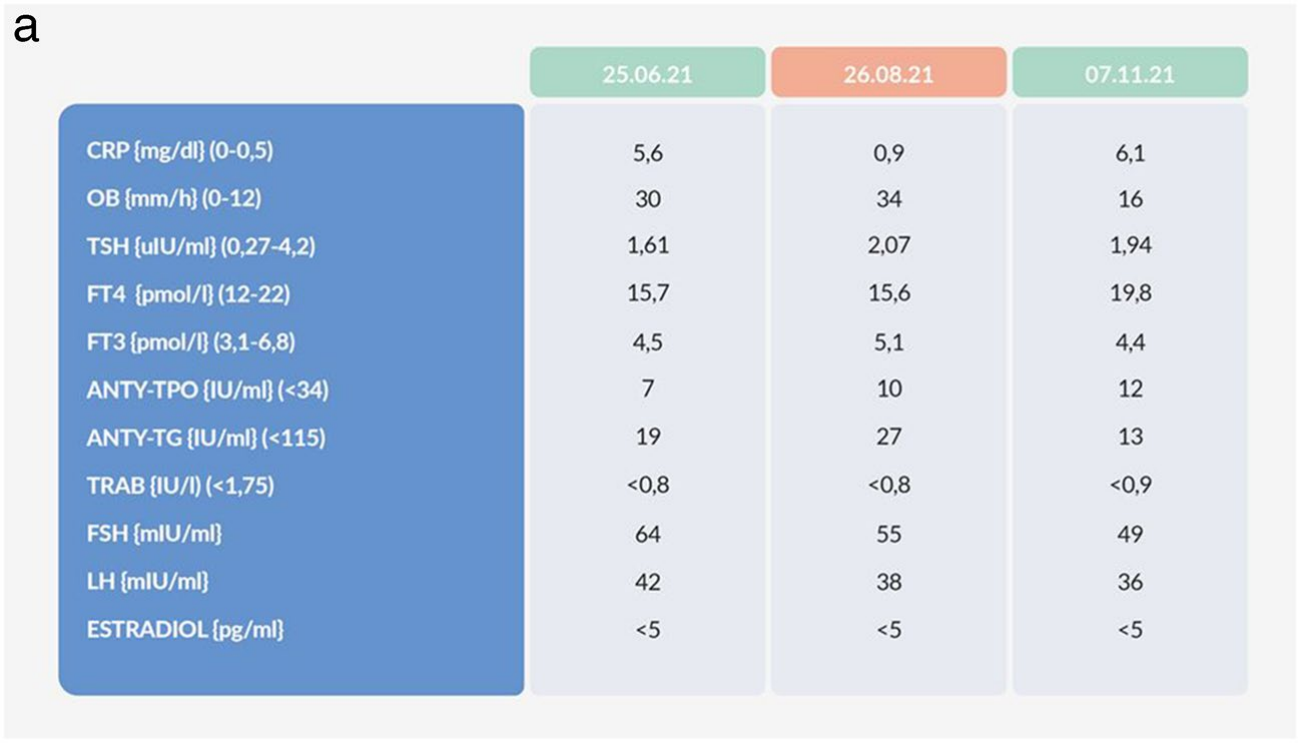

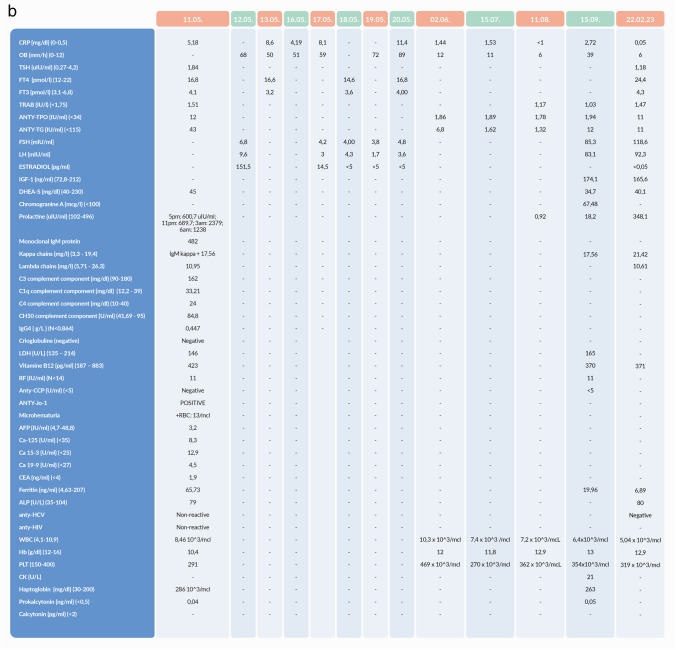
Summary of laboratory results

**Fig. 12:**
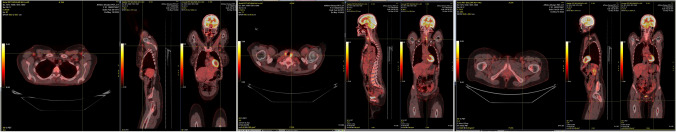
PET-CT with 18F-FDG: intense uptake of radiolabel in foci in the thyroid was visualized. The image may correspond to inflammatory lesions. PET-CT cannot exclude proliferative changes. In addition, axillary and inguinal lymphadenopathy was visualized bilaterally with mildly increased uptake of radiolabel- the PET-CT image requires differentiation between lymphoproliferative and reactionary lesions

**Fig. 13:**
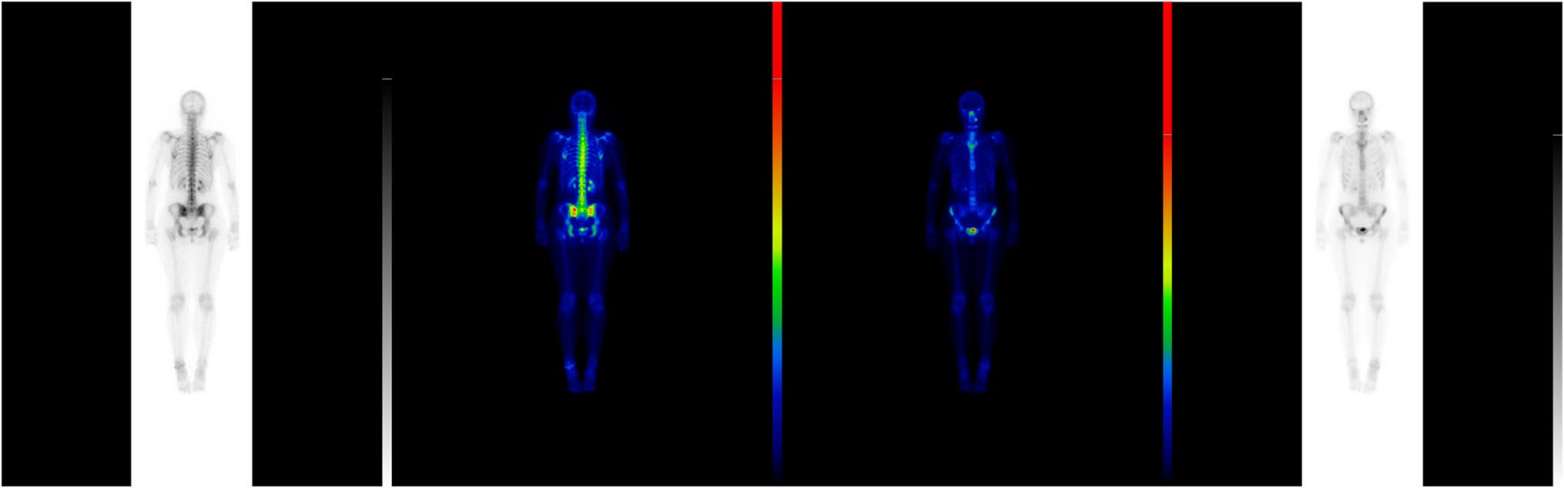
Bone scintigraphy (Tc99m MDP): increased tracer accumulation in the posterior part of the right rib 11

**Fig. 14:**
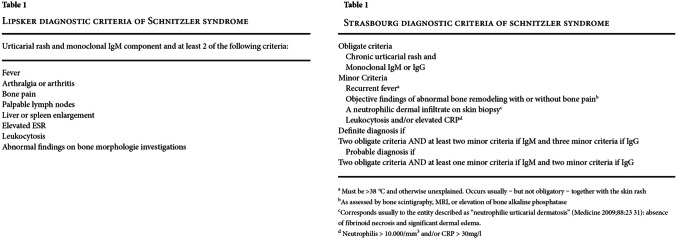
The patient desribed by us fulfills the Strasbourg and Lispker criteria for Schnitzler syndrome

Nevertheless, we had a feeling that our diagnostic approach in this case was not sufficient because, in spite of the surgical treatment, our patient still suffered from persistent fever, urticaria, and muscle pain, and presented elevated inflammatory markers. Therefore, we undertook a wide range of diagnostic procedures to search for another systemic disease. Laboratory tests showed M-protein in the IgM class, type kappa, amyloid AA, and ANA Jo1 (Fig. 11a and b). Bone marrow biopsy revealed discrete features of dysplasia and no infiltrations of lymphoma. The image of lymph nodes on FDG-PET scans might have suggested their reactive nature or their involvement in a lymphoproliferative process with a moderate tracer uptake (Fig. 12). Bone scintigraphy showed increased tracer uptake in the posterior part of the right 11th rib. In addition, some features of degenerative lesions in the left metatarsal were seen (Fig. 13). The fact that one disease (SAT) has been cured after thyroidectomy does not mean that the patient is cured. When signs and symptoms persisted after glucocorticoid discontinuation and considering the whole clinical picture, a decision was made to perform more thorough diagnostic investigations in this patient. The natural course of hypophysitis is unpredictable. In our patient, full recovery of hyperprolactinemia and pituitary transient edema was seen only during the administration of glucocorticoids and low-dose cabergoline. Fortunately, unlike in SAT, the hypophysitis did not relapse after the corticoid dose reduction. The patient’s postmenopausal status can increase the risk of osteoporosis and cardiovascular complications. In the future, long term follow-up will be mandatory to monitor for the development of hormonal deficits. Due to the presence of M-protein in the IgM class, type kappa, recurrent urticarial rashes, subfebrile temperatures, and bone lesions found on scintigraphy and following dermatological and rheumatological consultations, the patient was suspected of having Schnitzler syndrome: finally, she appeared to meet both the Strasbourg and Lipsker criteria for diagnosis of this disease entity (Fig. 14).Whether in SAT or in hypophysitis, skin lesions are not observed and monoclonal protein is not detected in the serum.

**Fig. 15:**
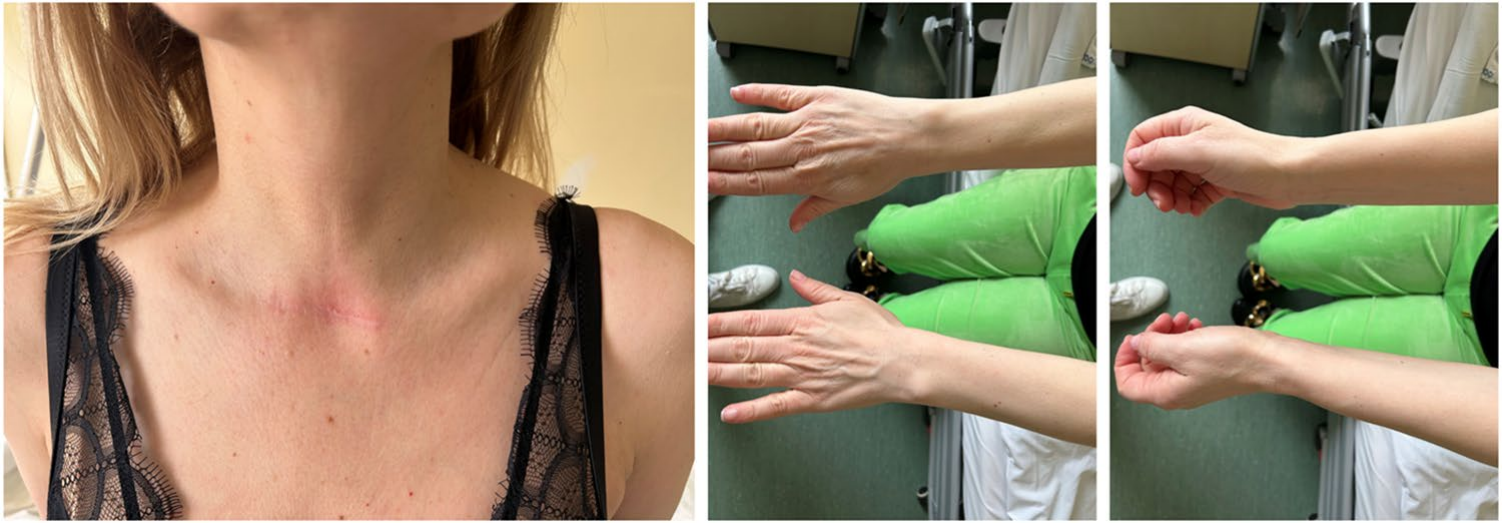
Resolution of skin lesions after anakinra treatment

The medical team decided to continue the ongoing treatment plan. The patient was discharged from hospital with the instruction to continue administration of IL-1 antagonist (anakinra), which is the first-line treatment for Schnitzler syndrome. The other medications prescribed included deflazocort at gradually reduced doses, cabergoline 0.5 mg/10 days, l-thyroxine 88 μg daily, calcium, and vitamin D. Following the IL-1 receptor antagonist (anakinra) therapy, a spectacular improvement was noted: all the symptoms subsided and inflammatory markers returned to normal (Fig. 15).

At further follow-up, the patient did not require any steroid therapy: the inflammatory process (represented by the presence of MGUS and elevated amyloid levels) induced over the 17 months by recurrent SAT as well as Schnitzler syndrome finally subsided thanks to the IL-1 antagonist treatment. Since there are reports in the literature of spontaneous remission of Schnitzler syndrome, further observation is needed to determine whether the patient needs to continue the therapy.

Obviously, continued screening for lymphoproliferative diseases will be necessary in this patient, especially for Waldenstrom macroglobulinemia, which develops in 15% of patients within 15–20 years of the onset of Schnitzler syndrome.

## Differential diagnosis

In the differential diagnosis of SAT, other causes of painful goiter should be considered, including rapidly growing thyroid cancers, lymphomas (including Hodgkin lymphoma) [65], metastases [66], hematomas, or Pneumocystis carini infection. Highly aggressive tumors (such as anaplastic carcinoma) rapidly infiltrate the surrounding tissues, including the neck muscles, and may cause symptoms similar to those of SAT. Thyroid abscess may also be difficult to differentiate from SAT and sometimes may develop as a result of infection of necrotic tissues in anaplastic carcinoma, as described by Lewiński et al. [6]. Prakash et al. reported a case of follicular cancer with clinical presentation that mimicked SAT as it manifested with hard and painful right lobe of the thyroid [66]. It must be kept in mind that good therapeutic response to glucocorticoids is not a diagnostic criterium for SAT since improvement with steroid therapy can also be seen in some cancers, metastatic lesions, and amyloidosis [67]. In the literature, while there are reports of systemic amyloidosis with symptoms of SAT, our patient did not present either renal dysfunction or cardiovascular disease and no amyloid deposits were found on histopathologic examination. Our patient underwent thorough diagnostic investigation for malignant diseases, including lymphoma, and the result was negative. Since SAT may develop as a paraneoplastic syndrome [68], diagnostic investigations in our patient were carried out via PET-FDG and trepanobiopsy. Moreover, we additionally considered amyloidosis, idiopathic urticaria, Sweet syndrome, and synthetase syndrome.

## Discussion and conclusions

In the group of patients with COVID-19 who later developed SAT, the female-to-male ratio was high, this being the opposite of what happens in a case series of hospitalized COVID-19 patients [68]. Studies of patients with SAT following COVID-19 reported that systemic general symptoms occurred in 85.2% of patients, neck pain in 92.6% of patients, and fever in 74.1% of patients [69]. Inflammatory markers were positive in 96% of patients. Anti-TG antibodies were detected in 3/11 patients, anti-TPO antibodies in 3/16, and TSH receptor antibodies (TRAb) were always negative [69]. In all reported cases of SAT following COVID-19 hyperthyroidism was observed. However, our patient remained euthyroid for the entire duration of her disease. In addition, according to the available meta-analyses, in 83.3% of cases the median time between COVID-19 and SAT was 30 days (IQR: 16 -32) [70]. In 12.5% of cases, COVID-19 and SAT occurred concurrently. Our patient developed SAT 12 weeks after COVID-19. The above presented data may suggest that the triggering factor for SAT development in our patient was COVID-19 vaccination. Meta-analyses have shown that SAT following COVID-19 vaccination develops after an average of 10 days (3–12 days) [71]: in our patient, the onset of SAT was 12 days after COVID-19 vaccination. The reported patient has a haplotype that suggests an increased genetic susceptibility to ASIA syndrome following SARS-CoV-2 vaccination. A study that compared 14 patients with vaccination-induced SAT versus a control group of 100 subjects has shown that the presence of the HLA-B35 and HLA-C*04:01 alleles was considerably more prevalent in patients with SAT following SARS-CoV-2 vaccination [37].

The etiology of hypophysitis is most likely autoimmune. Almost 30% of patients have a concomitant autoimmune disease. Moreover, in one of the most recent retrospective studies, antinuclear and anti-extractable nuclear antigens, including Jo-1 antibodies (as in our patient), were detected. Similarly to hypophysitis, Schnitzler syndrome is probably an acquired autoimmune disease, and we suspect that its development in our patient after COVID-19 vaccination was additionally influenced by increased genetic susceptibility to ASIA syndrome due to the presence of the HLA-B*35:01 and HLA-C*04:01 alleles. Whether the presence of the HLA-B*35:01, HLA-DQA1*02:01 and HLA-DQB1:05:02 alleles, which increase susceptibility to chronic urticaria, can also contribute to the development of Schnitzler syndrome requires further investigation.

A meta-analysis by Zhang [28] has shown that therapy with non-steroidal anti-inflammatory drugs (NSAIDs) versus glucocorticoids can reduce the risk of SAT development. However, this may be explained by the fact that patients treated with NSAIDs have a milder course of the disease. Evidence suggests that in patients at high risk of SAT recurrences, the treatment should be started with high-dose prednisone followed by its gradual dose reduction over a longer period of time: glucocorticoid withdrawal within 30 days can increase the risk of recurrence. The standard recommendation is to use prednisone 40 mg/day for 1—2 weeks and then gradually taper the dose. As reported in the literature, SAT recurrences are observed when prednisone dose is reduced down to 5–10 mg [28]; therefore, prolonged administration of the 10 mg dose is required [28]. In our patient, even this recommendation failed to be successful. Factors that increased the risk of SAT recurrences in our patient include the presence of haplotypes known to confer high risk for developing SAT, female sex, euthyroid status, and negative thyroid antibodies.

Total thyroidectomy is not a standard treatment of SAT. However, in view of the very aggressive course of the disease, its recurrent nature, and the presence of haplotype known to confer high risk for developing SAT, we decided to choose the most extreme treatment option, i.e., thyroidectomy, taking this decision due to the patient’s refractory recurrent SAT. According to the literature, thyroidectomy may be the final treatment of recurrent SAT. Until now only a little over 20 patients were reported to have undergone thyroidectomy because of SAT recurrences [63, 72]. Total thyroidectomy in recurrent SAT should be performed in tertiary reference hospitals by highly experienced surgeons given that there is a risk of complications, such as recurrent laryngeal nerve damage or hypoparathyroidism. Surgical treatment in a SAT patient may be challenging because of rock-hard consistency of the thyroid due to chronic inflammation. Follicular fibrosis may result in difficulties in dissecting the thyroid gland from surrounding tissues. Nevertheless, total thyroidectomy performed by an experienced surgeon is associated with low morbidity. In a case series of 10 patients, difficulties with thyroid mobilization were encountered only in one patient [73]. In a study of 17 patients, unilateral lobectomy was performed in four patients, subtotal thyroidectomy in 12 patients, and total thyroidectomy in one patient, with a low rate of complications [74]. There is a case report of a 76-year old male patient with recurrent SAT who underwent total thyroidectomy with no complications, with both recurrent laryngeal nerves and three parathyroid glands intact. Complete relief of pain was achieved postoperatively [75]. However, the patient presented in our case report is the first patient ever with the presence of haplotypes of high risk of SAT who underwent total thyroidectomy.

We should never leave any patient thinking that we have cured them: for example, unfortunately, the thyroidectomy performed in this patient improved only some of the patient’s signs and symptoms. What is noteworthy in the reported patient with recurrent SAT and Schnitzler syndrome following SARS-CoV-2 vaccination is steroid dependence. When the prednisolone dose was reduced, signs and symptoms of the disease with urticaria and SAT symptoms (fever, neck pain, and elevated CRP and ESR) have subsided. Thyroidectomy most probably reduced the autoimmune inflammatory process, and urticaria recurred when the prednisone dose was reduced down to 2.5 mg daily (versus the preoperative dose of 5 mg daily), while the fever returned when the prednisolone dose was reduced down to 5 mg daily (versus the preoperative dose of 10 mg daily). Furthermore, postoperative glucocorticoid discontinuation did not induce such a severe increase in inflammatory markers as had been observed before the surgery. Therefore, one of the benefits of the surgical treatment was the steroid sparing effect. Of course, another obvious benefit was alleviation of the severe neck pain that accompanied every SAT episode and decreased the patient’s quality of life. However, it is only the initiation of the causative treatment of Schnitzler syndrome, i.e. anakinra (IL-1 antagonist), that resulted in complete remission of all signs and symptoms. What is also interesting is the resorption of the pituitary inflammatory edema within 4 months of the onset of cabergoline and corticosteroid treatment and the specific gonadotropin pattern. Initially, gonadotropin levels were suppressed, while cabergoline treatment unblocked the gonadotropin axis and resulted in gonadotropin levels typical for postmenopausal women.

In our differential diagnosis, we considered other causes of pituitary edema, including IgG4-related hypophysitis: IgG4 antibodies were, however, negative and, apart from hyperprolactinemia, no other hormonal disorders were observed. Furthermore, the image of the lesion on MRI was not typical for sarcoidosis, histiocytosis, or Wegener’s granulomatosis.

It is very likely that the autoimmune disease, i.e., Schnitzler syndrome, affected both the thyroid gland – causing persistent subacute thyroiditis – and the pituitary gland – causing hypophysitis and its accompanying transient inflammatory edema. There is no doubt that the illness caused by COVID-19 infection and the vaccination against COVID-19 resulted in immunization of the body. The persistent nature of SAT was most likely caused by the rebound effect or the activation of the immune system (ASIA syndrome) following the vaccination or the infection with SARS-CoV-2.

The recurrence of SAT seems to occur not under continuous viral infection but after disappearance of immunity from a previous viral infection and after activation of immunity following a vaccination. In our patient, SAT represents a multistystem disease affecting the thyroid. SAT and hypophysitits are most likely to represent a nonspecific inflammatory response of the thyroid to one of a variety of antigens. Urticaria and SAT may be mediated by cytokines, with impaired T cell function. Urticaria may also develop as a hypersensitivity reaction to a systemic inflammatory response triggered by SAT.

In our case, the 17-month course of refractory recurrent steroid-resistant SAT in a female patient was well established and the observed signs and symptoms, including recurrent pain with fever and chronic urticaria, fully met the diagnostic criteria of Schnitzler syndrome. Total thyroidectomy, performed by an experienced surgeon, seems to be a safe and effective treatment for recurrent SAT.

To the best of our knowledge, this is the first case report ever published in the literature that presents a patient with concomitant SAT, hypophysitis, and Schnitzler syndrome and also the first case report of Schnitzler syndrome, hypophysitis, and SAT induced by vaccination against COVID-19. Furthermore, it is the first case report of coexistence of Schnitzler syndrome and recurrent SAT in a patient with strong genetic susceptibility to SAT and chronic urticaria due to the presence of three alleles associated with a high risk of development of SAT and the presence of alleles associated with a high risk of development of CUA. It is also the first case report of recurrent SAT induced by SARS-CoV-2 vaccination and the first case report of total thyroidectomy in a patient with SAT following SARS-CoV-2 vaccination.

**Fig. 16:**
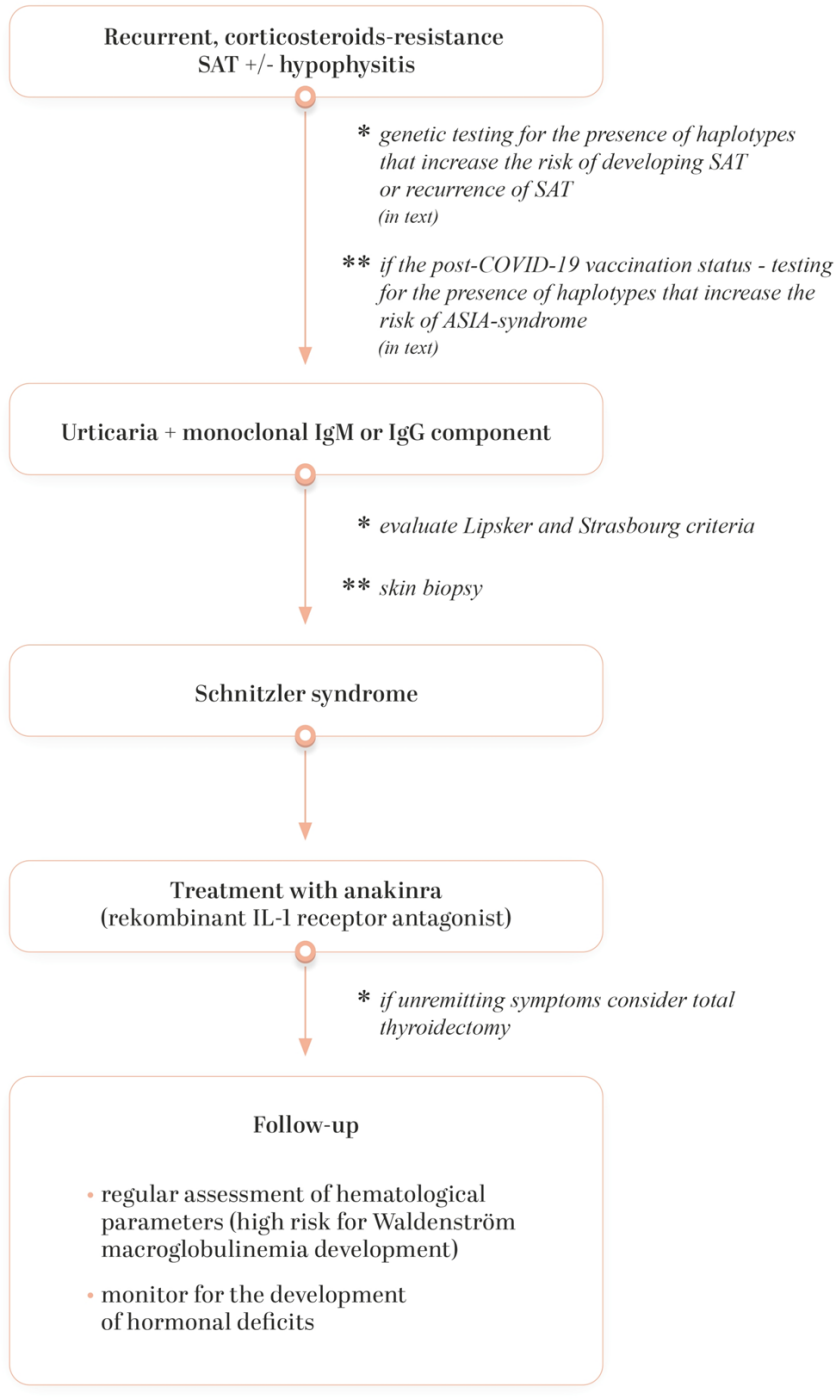
Proposed diagnostic and therapeutic algorithm

To conclude, in the event of recurrent glucocorticoid-resistant SAT or hypophysitis with accompanying urticaria, the next step in the diagnostic approach should be an investigation into the potential presence of IgM monoclonal protein, which leads to the suspicion of Schnitzler syndrome (Fig. 16). Additionally, it is useful to perform genetic testing so as to possibly confirm the presence of haplotypes associated with high risk of SAT and ASIA syndrome following COVID-19 vaccination. When the Lipsker and Strasbourg criteria are confirmed and a skin biopsy is performed, the patient should be started on treatment, which involves the administration of an IL-1 antagonist (anakinra). The patient’s follow-up should include a search for lymphoproliferative diseases, especially for Waldenstrom macroglobulinemia, which develops in 15% of patients within 15–20 years of the onset of Schnitzler syndrome. Moreover, long-term follow-up is mandatory to monitor for the development of hormonal deficits, which may occur following hypophisitis.

## Data Availability

Data sharing is not applicable to this article as no datasets were generated or analyzed during the current study.
